# Analysis of the complete genome sequence of *Bradyrhizobium diazoefficiens* 172S4, a highly efficient nitrogen-fixing symbiont of soybeans, reveals large-scale genomic inversion

**DOI:** 10.1128/mra.00889-25

**Published:** 2025-11-05

**Authors:** Eden S. P. Bromfield, Sylvie Cloutier

**Affiliations:** 1 Agriculture and Agri-Food Canada 6337, Ottawa, Ontario, Canada; Indiana University Bloomington, Bloomington, Indiana, USA

**Keywords:** *Bradyrhizobium diazoefficiens *172S4, complete genome sequence, large chromosomal inversion, nitrogen fixation, nitrous oxide reductase genes, hydrogen uptake genes, Canada, soybeans, symbiosis

## Abstract

We announce the complete genome sequence of *Bradyrhizobium diazoefficiens* 172S4, a highly efficient nitrogen-fixing symbiont isolated from a soybean root nodule. The chromosome (~9.1 Mb) exhibits a large inversion (~5.3 Mb) and encodes genes for symbiosis, nitrogen fixation, N_2_O reduction, and hydrogen uptake, highlighting the potential of 172S4 for sustainable agriculture.

## ANNOUNCEMENT

Soybeans are routinely inoculated with nitrogen-fixing *Bradyrhizobium* strains to reduce the need for synthetic nitrogen fertilizers. In previous work (1), soybeans served as trap plants to isolate beneficial bradyrhizobia from root-zone soils of native Canadian legumes to identify potential new inoculant strains. One such isolate, designated *Bradyrhizobium diazoefficiens* strain 172S4, was isolated from a surface-sterilized soybean nodule following inoculation with soil collected from *Amphicarpaea bracteata* (coordinates 46°41′4.83″ N and 71°44′28.05″ W). In that study (1), strain 172S4 demonstrated superior nitrogen fixation efficiency in symbiosis with soybeans compared to the widely used inoculant strain, *B. diazoefficiens* USDA110^T^ (2).

Novel strain 172S4 was cultured on yeast extract mannitol agar for 7 days at 28°C (1). Genomic DNA was extracted with the Wizard SV genomic DNA purification system (Promega, USA), purified with the DNeasy PowerClean Pro kit (Qiagen, Germany), sheared to ~20 kb, and prepared with the SMRTbell Template Prep Kit 1.0 (PacBio) for sequencing on the PacBio RS II platform at the Genome Quebec Innovation Center (Canada), generating 107,050 subreads (mean 13,188 bp; N50 13,714 bp) at ~138× coverage. Subreads were quality-filtered and adapter sequences trimmed and error-corrected prior to *de novo* assembly using HGAP in SMRT Analysis (v.2.3) (3, 4). The genome was circularized and rotated using Circlator (v.1.5.5) (5), quality assessed with CheckM (v.1.2.3) (6), and annotated using the National Center for Biotechnology Information Prokaryotic Genome Annotation Pipeline (v.6.6) (7).

Table 1 shows the characteristics of the genome sequence of 172S4 compared with reference strain USDA110^T^ (8). The genome of 172S4 consists of a single circular chromosome of ~9.1 Mb with G + C content of 64%. A symbiosis island (~0.7 Mb) was identified, containing key genes for nodulation (*nod*, *nol*, and *noe*), nitrogen fixation (*nif* and *fix*), and a Type III secretion system. Additional features include a complete *nos* gene cluster encoding nitrous oxide reductase, which facilitates the reduction of N_2_O (a potent greenhouse gas) to benign dinitrogen; hydrogen uptake (*hup*) genes, implicated in enhancing the efficiency of nitrogen fixation by recycling hydrogen formed as a by-product of nitrogenase activity (9); a CRISPR-Cas system detected using CRISPR-CasFinder (v1.1.2) (10); and an intact prophage (23,387 bp) detected using PHASTEST (v.3.0) (11).

**TABLE 1:** Characteristics of the complete genome sequence of *Bradyrhizobium diazoefficiens* 172S4 relative to reference strain *Bradyrhizobium diazoefficiens* USDA 110^T^

Characteristics	172S4	USDA 110^T^
Genome accession no.	CP050064	BA000040
Genome size (bp)	9,142,067	9,105,828
Genes (total)	8,571	8,577
CDSs (total)	8,513	8,271
G + C content (%)	64.0	64.1
No. of rRNA operons (5S, 23S, 16S)	1	1
tRNAs	51	53
Coordinates (base pair positions) of		
Symbiosis island	7,649,953–8,325,197	1,681,000–2,362,000
Nodulation genes (*nodD2D1YABCSUIJ*, *nolAKNOYZUV*, and *noeDEIL*)^*a*^	7,768,097–8,225,257	1,788,235–2,243,506
Nitrogen fixation genes (*nifHDKENA* and *fixABCX*)	8,076,927–8,104,183	1,907,825–1,935,084
Type III secretion system genes (*rhcC1C2JNQRSTUV*)	8,018,958–8,059,652	1,955,237–1,974,703
Hydrogen uptake genes (*hupNCUVSLCDFGHIJK*)	2,234,636–2,253,781	7,630,443–7,649,197
Nitrous oxide reductase genes (*nosRZDFYLX*)	579,116–588,054	324,547–333,495
Prophage	4,818,594–4,841,981	nd^*b*^
CRISPR-CAS	7,337,444–7,337,544	nd^*b*^

^*a*^ *noeE* gene not detected in 172S4.

^*b*^ nd, not detected.

The average nucleotide identity (FastANI v.1.34) (12) and digital DNA-DNA hybridization (dDDH v.4.02) (13) values for strain 172S4 versus USDA 110^T^ are 98.7% and 89.4% (confidence interval: 87.1%–91.4%), confirming assignment of 172S4 to the species *B. diazoefficiens*.

**Fig 1:**
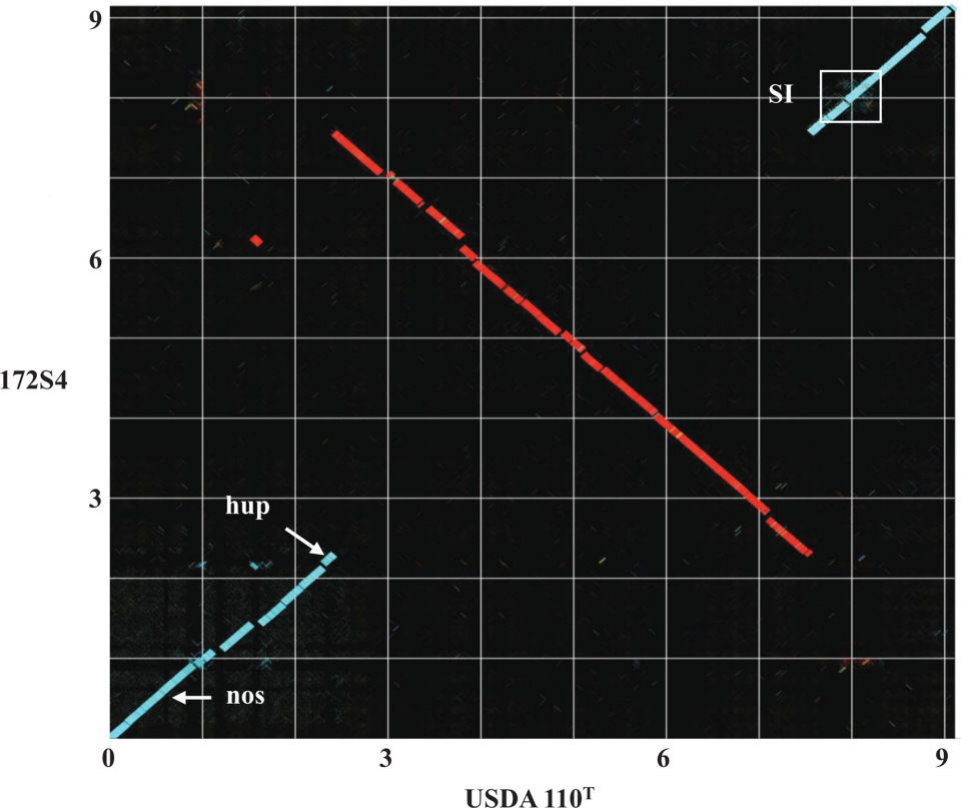
Linear comparison of the genome sequences of *Bradyrhizobium diazoefficiens* 172S4 (*y* axis) relative to reference strain, *Bradyrhizobium diazoefficiens* USDA110^T^ (*x* axis). The scale is shown in megabases. The inverted chromosomal region (~5.3 Mb) is shown in red. The locations of the symbiosis island (SI), hydrogen uptake (hup) genes, and nitrous oxide reductase (nos) genes are indicated in white.

A linearized comparison of the genome sequence of 172S4 with USDA110^T^ was performed using Genome Matcher (v.3) (14). Sequences were oriented so that they had the same directionality and the dnaA gene as starting point. The comparison (Fig. 1) revealed a large ~5.3 Mb chromosomal inversion in 172S4 relative to USDA110^T^. Similar large-scale rearrangements have been reported in other bacteria (15). Notably, genes responsible for symbiosis, nitrogen fixation, hydrogen metabolism, and denitrification in 172S4 are located outside the inverted region, suggesting preservation of their functional phenotypes.

These findings further the understanding of the genomic organization of efficient symbiotic nitrogen-fixing *Bradyrhizobium* strains showing potential as elite soybean inoculants.

## Data Availability

The whole genome shotgun project for *Bradyrhizobium diazoefficiens* strain 172S4 was deposited at DDBJ/ENA/GenBank as accession number CP050064. Raw PacBio data were deposited in the National Center for Biotechnology Information Sequence Read Archive as SRX7909064 with BioProject accession number PRJNA612209. Sequence accession numbers for the 16S rRNA gene are KP768822 (Sanger sequence) and CP050064 (complete genome sequence).
