# Correction for Bromfield and Cloutier, “Analysis of the complete genome sequence of *Bradyrhizobium diazoefficiens* 172S4, a highly efficient nitrogen-fixing symbiont of soybeans, reveals large-scale genomic inversion”

**DOI:** 10.1128/mra.00627-26

**Published:** 2026-07-20

**Authors:** Eden S. P. Bromfield, Sylvie Cloutier

## AUTHOR CORRECTION

Volume 14, no. 12, e00889-25, 2025, https://doi.org/10.1128/mra.00889-25. Announcement, paragraph 3: The following text was reported in error and should be disregarded: “a CRISPR-Cas system detected using CRISPR-CasFinder (v1.1.2) (10);”

Table 1: The CRISPR-Cas sequence entry under the 172S4 column (“7,337,444–7,337,544”) was reported in error and should be disregarded.

Data Availability: The following sentence should be added to the end of the section: “*Bradyrhizobium diazoefficiens* strain 172S4 has been deposited in the BCCM/LMG Bacteria Collection, University of Ghent, Belgium, under the culture collection number LMG 34613.”

References: Reference 10 should be deleted.

These modifications ensure the accuracy and completeness of the public record for this genome announcement. They do not alter the overall scientific conclusions of the paper.

